# Airway *β*-Defensin-1 Protein Is Elevated in COPD and Severe Asthma

**DOI:** 10.1155/2015/407271

**Published:** 2015-10-19

**Authors:** Katherine J. Baines, Thomas K. Wright, Jodie L. Simpson, Vanessa M. McDonald, Lisa G. Wood, Kristy S. Parsons, Peter A. Wark, Peter G. Gibson

**Affiliations:** ^1^Priority Research Centre for Asthma and Respiratory Diseases, Hunter Medical Research Institute, The University of Newcastle, New Lambton Heights, NSW 2305, Australia; ^2^Department of Respiratory and Sleep Medicine, John Hunter Hospital, New Lambton Heights, NSW 2305, Australia

## Abstract

*Background*. Innate immune antimicrobial peptides, including *β*-defensin-1, promote the chemotaxis and activation of several immune cells. The role of *β*-defensin-1 in asthma and chronic obstructive pulmonary disease (COPD) remains unclear. *Methods*. Induced sputum was collected from healthy controls and individuals with asthma or COPD. *β*-defensin-1 protein in sputum supernatant was quantified by ELISA. Biomarker potential was examined using receiver operating characteristic curves. *β*-defensin-1 release from primary bronchial epithelial cells (pBECs) was investigated in culture with and without cigarette smoke extract (CSE). *Results*. Airway *β*-defensin-1 protein was elevated in COPD participants compared to asthma participants and healthy controls. Inflammatory phenotype had no effect on *β*-defensin-1 levels in asthma or COPD. *β*-defensin-1 protein was significantly higher in severe asthma compared to controlled and uncontrolled asthma. *β*-defensin-1 protein could predict the presence of COPD from both healthy controls and asthma patients. Exposure of pBECs to CSE decreased *β*-defensin-1 production in healthy controls; however in pBECs from COPD participants the level of *β*-defensin-1 remanied unchanged. *Conclusions*. Elevated *β*-defensin-1 protein is a feature of COPD and severe asthma regardless of inflammatory phenotype. *β*-defensin-1 production is dysregulated in the epithelium of patients with COPD and may be an effective biomarker and potential therapeutic target.

## 1. Introduction

Asthma and chronic obstructive pulmonary disease (COPD) are chronic inflammatory airway diseases, characterised by airflow limitation obstruction that is reversible in asthma yet, under current therapeutics, progressive and not completely reversible in COPD. The global burden imposed by these diseases is considerable [1]. Asthma and COPD are recognized for their heterogeneous nature, particularly in regard to the type of inflammation present [2]. Four major phenotypes (eosinophilic, neutrophilic, paucigranulocytic, and mixed granulocytic) have been described and characterised by the proportions of eosinophils and neutrophils [2, 3]. These inflammatory phenotypes are associated with differences in disease severity and response to corticosteroids [4, 5], yet their underlying biology remains poorly understood [6, 7].

The migration and activity of eosinophils and neutrophils are influenced by a range of host factors, including a class of antimicrobial peptides also known as alarmins. These antimicrobial peptides are small (<100 amino acids) proteins, which play an important role in influencing and modulating the immune response through the receptor-mediated chemotaxis and activation of a range of innate and adaptive immune cells [8]. Defensins and cathelicidin constitute key alarmin families in humans [8]. The *α*- and *β*-defensins constitute the two major classes of human defensins, classified based on the differential organisation of six cysteine motifs [9]. *β*-defensin-1 is constitutively expressed by the epithelial cells of the respiratory tract and is both broadly antimicrobial and able to influence the immune response [10].

*β*-defensin-1 polymorphisms have been associated with both asthma [11, 12] and COPD [13, 14]. Increased gene expression of *β*-defensin-1 has been observed in bronchial epithelium and BAL fluid cells in COPD and was negatively associated with lung function and airflow limitation [15]. *β*-defensin-1 protein, however, has yet to be examined in either asthma or COPD. This study characterises the protein levels of *β*-defensin-1 in induced sputum samples in both asthma and COPD compared with healthy controls. We investigate the relationship of *β*-defensin-1 to inflammatory phenotypes and disease severity. To understand the potential source of aberrant *β*-defensin-1 expression, we also investigate its production in primary bronchial epithelial cells exposed to cigarette smoke extract in culture. We hypothesised that protein expression would be greater in asthma and COPD, associated with disease severity and inflammatory phenotype, and altered in epithelial cells after cigarette smoke exposure.

## 2. Methods

### 2.1. Study Design and Sputum Analysis

Adults with COPD and asthma were recruited from the John Hunter Hospital Ambulatory Care Clinic, NSW, Australia. Participants with COPD (*n* = 43 and *n* = 10 for the primary bronchial epithelial cell (pBEC) study) had a diagnosis of COPD and postbronchodilator FEV_1_/FVC < 70%. Participants with asthma (*n* = 94) were diagnosed according to American Thoracic Society guidelines based upon current (past 12 months) episodic respiratory symptoms, doctor's diagnosis, and demonstrated evidence of airway hyperresponsiveness to hypertonic saline. Healthy controls (*n* = 28 and *n* = 4 for the pBEC study) were nonsmokers and had FEV_1_ > 80% predicted and were recruited by advertisement. Exclusion criteria included a respiratory tract infection, exacerbation of respiratory disease or change in maintenance therapy in the past month, and current smoking (except for the pBEC study where current smoking in the COPD group was not exclusion criteria). All participants gave written informed consent and the Hunter New England Area Health Service and The University of Newcastle Research Ethics Committees approved this study.

Spirometry (KoKo PD Instrumentation, Louisville, Colorado, USA) and sputum induction with hypertonic saline (4.5%) were performed in participants with FEV_1_ > 1.3 L and with 0.9% saline in participants whose FEV_1_ was below this level. The protocol specified a fixed sputum induction time of 15.5 minutes. For inflammatory cell counts, selected sputum was dispersed using dithiothreitol (DTT), suspension was filtered, and a total cell count and cell viability count were performed. Cytospins were prepared and stained (May-Grunwald Giemsa) and a differential cell count was obtained from 400 nonsquamous cells.

### 2.2. Measurement of *β*-Defensin-1 Protein

The concentration of *β*-defensin-1 was determined by ELISA (100-240-BD1, Alpha Diagnostic International, San Antonio, Texas, USA) as per manufacturer's instructions. The standard curve ranged from 50 to 800 pg/mL. The measurement of *β*-defensin-1 protein in sputum supernatant was validated by determining the following: the inhibitory effect of DTT, matrix effects of sample dilution, and the recovery of spiked protein. DTT had an insignificant effect on the standard curve. Matrix effects were minimised when samples were diluted 1/10. On average, 104% of spiked *β*-defensin-1 protein (*n* = 6) was recovered.

### 2.3. Disease Classifications

The granulocyte cut-offs to determine inflammatory phenotype used were ≥3% for sputum eosinophils and ≥61% for sputum neutrophils [2]. Severe asthma subjects had uncontrolled asthma (measured by Juniper Asthma Control Questionnaire (ACQ), score ≥ 1) and/or poor lung function (FEV_1_% predicted ≤80 and FEV_1_/FVC% ≤ 70) despite prescription of high-dose inhaled corticosteroids (ICS) (>1000 *μ*g beclomethasone equivalents per day) in combination with long acting *β*-agonists [16]. If participants had poor lung function or poor symptom control but did not meet the treatment requirements for severe asthma, they were classified as uncontrolled asthma. Controlled asthma was defined as normal lung function (FEV_1_% predicted > 80 and/or FEV_1_/FVC% > 70) and controlled symptoms (ACQ score < 1). COPD severity was defined according to the global obstructive lung disease (GOLD) initiative stage [17].

### 2.4. Primary Bronchial Epithelial Cell (pBEC) Culture

Cigarette smoke extract (CSE) was prepared by bubbling smoke from one filterless Kentucky research cigarette, 3R4F containing 9.5 mg tar and 0.8 mg nicotine, through 10 mL of cell culture medium Bronchial Epithelial Basal Medium (BEBM, Lonza) at a speed of 5 minutes per cigarette and used in the following cell culture experiments immediately. Human pBECs were obtained by endobronchial brushing during fibre-optic bronchoscopy and cultured as described previously [18]. pBECs were maintained in Bronchial Epithelial cell Growth Medium (Lonza). Cells were seeded onto placental collagen (Sigma) coated 24-well plates (Nunclon) and used at passage 2 once they reached 80% confluency. After exposure to 1% CSE the pBECs were maintained in BEBM (Lonza) containing 1x insulin, transferrin, and sodium selenite liquid media supplement (Sigma). We have previously determined that this concentration of CSE via dose response curves causes minimal toxicity to the cells whilst still inducing an immune response. All cells were grown at 37°C with 5% CO_2_ in air. Cell culture supernatant was collected at 24 hrs for assessment of *β*-defensin-1 protein.

### 2.5. Statistical Analysis

Clinical and cell count data were analysed using Stata/IC 11.1 (Stata Corporation, College Station, Texas, USA) and GraphPad Prism 5.0 (GraphPad Software, Inc., California, USA) and reported as mean (SD) for normally distributed data and median (Q1 and Q3) for nonparametric data. For normally distributed data, *t*-test or ANOVA test with a Bonferroni post hoc test to adjust for multiple comparisons was applied. For nonparametric data, a Mann-Whitney test or Kruskal-Wallis test with Dunn's post hoc test to adjust for multiple comparisons was applied. Statistical comparisons for categorical data were made using a *χ* ^2^ test. Associations between variables were assessed using Spearman correlation coefficients. Receiver operating characteristic (ROC) curves were generated and the area under the curve (AUC) was calculated to assess the relationship between the presence of COPD and *β*-defensin-1 levels.

## 3. Results

### 3.1. Clinical Features and Inflammatory Cells in Asthma Participants, COPD Patients, and Healthy Controls

Clinical details and inflammatory cell are detailed in Table 1. Participants with COPD were moderate to severe (with 2 mild and 2 very severe), were significantly older, had a greater prevalence and smoking history, and a higher daily dose of ICS. Participants with asthma had a significantly higher BMI. As expected, FEV_1_% predicted and the FEV_1_/FVC were significantly lower in asthma subjects than in healthy controls and lower in COPD subjects than in both asthma subjects and healthy controls. Changes in inflammatory cells were present in asthma and COPD, including increases in total cell count, neutrophils, and eosinophils.

### 3.2. Airway *β*-Defensin-1 in Asthma Subjects, COPD Subjects, and Healthy Controls

**Figure 1:**
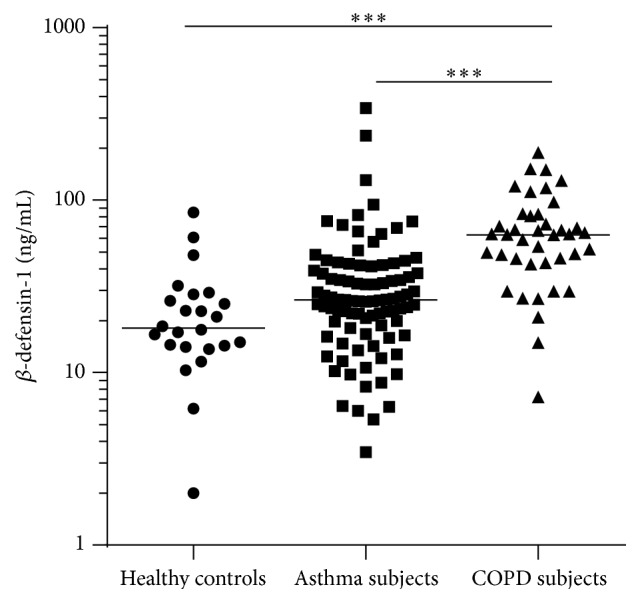
*β*-defensin-1 in airway disease. The airway expression of *β*-defensin-1 protein is elevated in participants with COPD compared to asthma patients and healthy controls (Kruskal-Wallis test; *p* < 0.001). The bars represent the median of each group.  ^*∗∗∗*^Dunn's post hoc testing *p* < 0.001.

**Figure 2:**
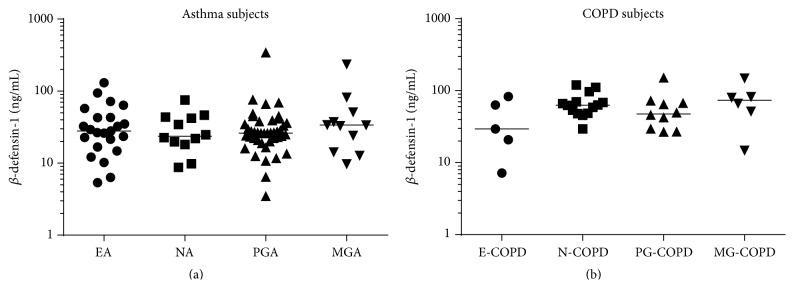
*β*-defensin-1 in inflammatory phenotypes. The airway expression *β*-defensin-1 protein is not different between inflammatory phenotypes of (a) asthma and (b) COPD. The bars represent the median of each group. EA = eosinophilic asthma; NA = neutrophilic asthma; PGA = paucigranulocytic asthma; MGA = mixed granulocytic asthma; E-COPD = eosinophilic COPD; N-COPD = neutrophilic COPD; PG-COPD = paucigranulocytic COPD; MG-COPD = mixed granulocytic COPD.

**Figure 3:**
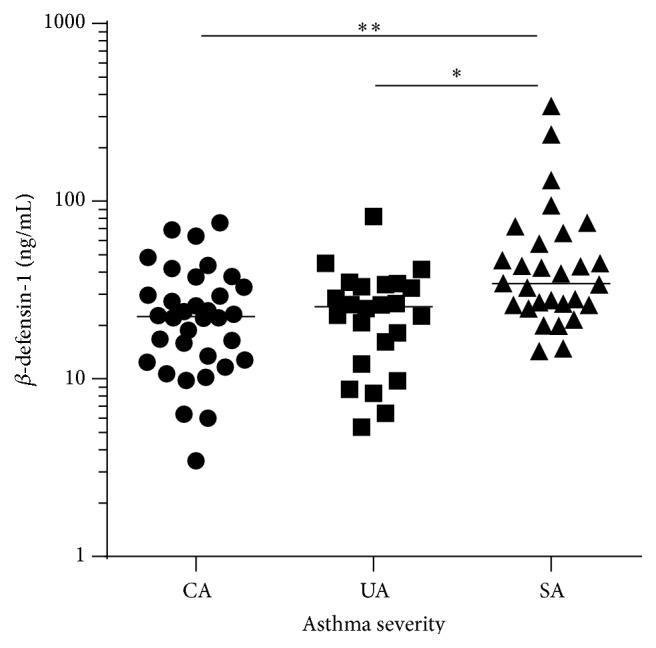
*β*-defensin-1 and asthma severity. The airway expression of *β*-defensin-1 protein is higher in severe asthma (SA; *n* = 29) compared with controlled asthma (CA, *n* = 34) and uncontrolled asthma (UA, *n* = 24) (Kruskal-Wallis test; *p* = 0.002). The bars represent the median of each group.  ^*∗*^Dunn's post hoc testing *p* = 0.003 versus controlled asthma.  ^*∗∗*^Dunn's post hoc testing *p* = 0.015 versus uncontrolled asthma.

**Figure 4:**
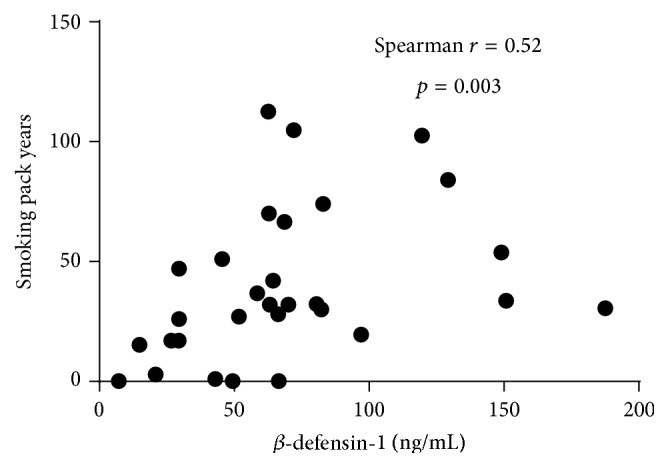
*β*-defensin-1 and smoking history in COPD. The airway expression of *β*-defensin-1 protein is correlated with pack years smoked.

*β*-defensin-1 protein in sputum was significantly higher in COPD subjects (median (q1 and q3): 63.0 (43.6 and 81.9)  ng/mL) than in asthma subjects (26.3 (18.3 and 40.8) ng/mL) and healthy controls (18.2 (14.2 and 27.8)  ng/mL; Figure 1). There was no significant difference in *β*-defensin-1 protein between inflammatory phenotypes of asthma or COPD (Figure 2). *β*-defensin-1 protein was significantly higher in those with severe asthma (Figure 3) but not different between COPD GOLD stages (data not shown). Airway *β*-defensin-1 protein level was correlated with smoking history (pack years smoked) in COPD subjects (Spearman *r* = 0.52 and *p* = 0.003, Figure 4) but not in asthma subjects or healthy controls. Airway *β*-defensin-1 protein level was weakly correlated with ICS dose in asthma (Spearman *r* = 0.31 and *p* = 0.006) but not in COPD, most likely reflecting the relationship to asthma severity. There was no correlation between airway *β*-defensin-1 protein and age of the participants.

**Figure 5:**
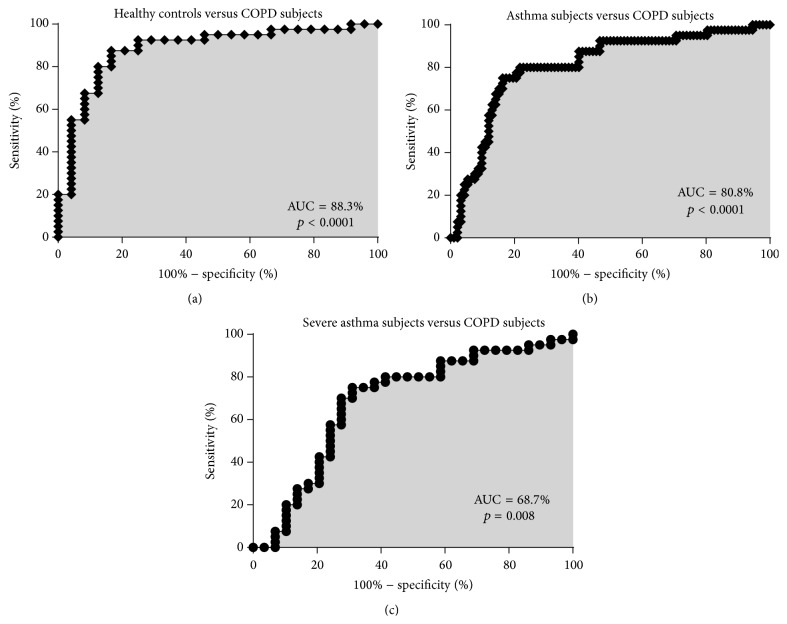
*β*-defensin-1 as a biomarker. Airway expression of *β*-defensin-1 can distinguish COPD from (a) healthy controls, (b) asthma patients, and (c) severe asthma patients.

Airway *β*-defensin-1 protein was predictive of COPD from healthy controls with an accuracy (AUC) of 88.3% (95% CI: 79.3–97.4%; Figure 5(a)). The best cut-off point for *β*-defensin-1 level to predict COPD from healthy controls was >29.3 ng/mL, with a sensitivity of 87.5%, specificity of 83.3%, and a positive likelihood ratio of 5.3. Airway *β*-defensin-1 protein was predictive of COPD from asthma with an accuracy (AUC) of 80.8% (95% CI: 72.5–89.1%; Figure 5(b)). The best cut-off point for *β*-defensin-1 level to predict COPD from asthma was >42.1 ng/mL, with a sensitivity of 80.0%, specificity of 78.3%, and a positive likelihood ratio of 3.7. Airway *β*-defensin-1 protein was predictive of COPD from severe asthma with an accuracy (AUC) of 68.7% (95% CI: 55.2–82.2%, Figure 5(c)). The best cut-off point for *β*-defensin-1 level to predict COPD from severe asthma was >47.3 ng/mL, with a sensitivity of 70.0%, specificity of 72.4%, and a positive likelihood ratio of 2.5.

### 3.3. Epithelial Cell Production of *β*-Defensin-1

**Figure 6:**
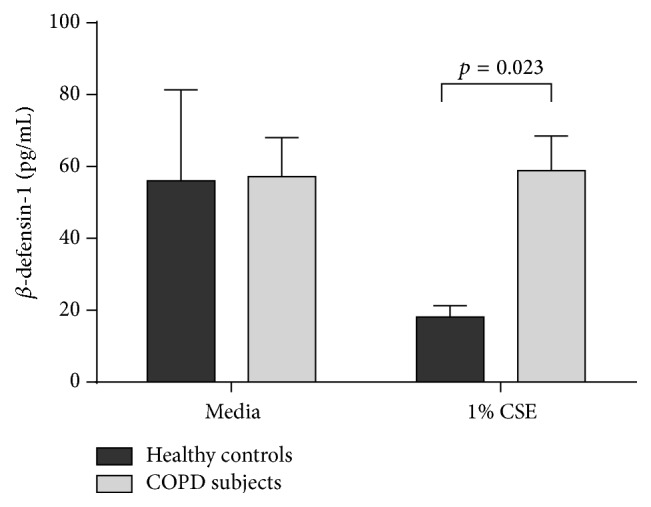
Production of *β*-defensin-1 from pBECs in response to cigarette smoke extract. The level of *β*-defensin-1 release is lowered in response to cigarette smoke in healthy pBECs (*n* = 4) but retained in pBECs from subjects with COPD (*n* = 10).

The level of *β*-defensin-1 released from untreated media control pBECs was similar between healthy controls and participants with COPD. However, upon stimulation with cigarette smoke extract (CSE) *β*-defensin-1 production was reduced by a mean of 2.8-fold in healthy control pBECs, whereas COPD pBECs continued to produce the same level of *β*-defensin-1 (mean −2.8-fold versus 0.1-fold, *p* = 0.003).  Figure 6 shows the levels of *β*-defensin-1 in culture, further illustrating this point, with the COPD group releasing significantly more *β*-defensin-1 than the healthy control pBECs after 1% CSE stimulation.

## 4. Discussion

To the authors' knowledge, this was the first study to examine *β*-defensin-1 protein in sputum in either asthma or COPD. We report an elevated level of *β*-defensin-1 protein in COPD and severe asthma, with no relationship to inflammatory phenotype. Airway levels of *β*-defensin-1 were correlated with smoking history in COPD. Airway levels of *β*-defensin-1 could significantly discriminate the presence of COPD from both asthma subjects and healthy controls. Production of *β*-defensin-1 was reduced in healthy pBECs but maintained in COPD pBECs after CSE exposure, which may account for the persistent and heightened *β*-defensin-1 levels.

Factors driving persistent inflammation in asthma and COPD, contributing to worsening of symptoms and lung function, are of interest to increase knowledge of underlying mechanisms and identify potential novel treatment targets. A class of multifunctional antimicrobial proteins known as alarmins, of which the defensins and the cathelicidins constitute two of the major families, may be of importance. Human *β*-defensin-1 is a small cationic peptide that is expressed constitutively by the epithelial cells of the respiratory tract. Research into the functions of *β*-defensin-1 has primarily centred around its antimicrobial properties; however other functions have been reported [19]. *β*-defensin-1 has immunomodulatory effects, promoting the activation and maturation of monocyte derived dendritic cells through upregulation of cell surface expression of costimulatory molecules and maturation markers, as well as the promotion of proinflammatory cytokine production [20]. By engaging a number of cell surface receptors including CCR6, *β*-defensin-1 promotes the chemotaxis of immature dendritic cells and T cells [21]. Through these immunomodulatory functions, *β*-defensin-1 may influence the pathogenesis of COPD, by promoting T cell and dendritic cell mediated inflammation.

Polymorphisms in the gene encoding *β*-defensin-1 (*DEFB1*) have been shown to affect the concentrations of *β*-defensin-1 protein detected in saliva, indicating that these gene polymorphisms do influence expression and therefore could modify innate immune responses [22], and may influence disease susceptibility. DEFB1 gene polymorphisms have been associated with both asthma [11, 12] and COPD susceptibility [13, 14]. Additionally, increase in* DEFB1* gene expression in bronchial epithelial and BAL fluid cells of patients with COPD is negatively correlated with FEV_1_% predicted and FEV_1_/FVC [15]. The distinct increase in *β*-defensin-1 protein seen in the current study supports a model in which *β*-defensin-1 is dysregulated in COPD and severe asthma. Additionally, we found a weak correlation between *β*-defensin-1 and ICS dose in asthma. Long term corticosteroid treatment can lead to changes in the balance between innate and adaptive immune responses, including cell migration and the chemokine network in macrophages [23]. Further study is required to determine the effects of long term ICS treatment on *β*-defensin-1 levels.

The development of new diagnostic tools and treatments for COPD has not kept pace with understanding of the disease [24]. As an airway sample, sputum provides appealing means to study potential biomarkers as it contains a multitude of inflammatory mediators involved in COPD disease processes [25]. This study also shows that *β*-defensin-1 protein levels in the sputum supernatant may be a useful biomarker for the detection of COPD. *β*-defensin-1 was able to distinguish COPD from both asthma subjects and healthy controls with high levels of accuracy. For many years, spirometry and clinical symptoms have been the predominant tools to manage COPD; however this approach is not optimal [26]. Sputum eosinophils and exhaled nitric oxide can predict eosinophilic inflammation in airways disease [24] but do not differentiate asthma and COPD. Blood biomarkers including C-reactive protein, IL-6, and fibrinogen can detect a systemic inflammatory component of COPD, important in predicting patients at risk of exacerbations [27], but these markers are not specific to COPD and have been determined to be elevated in the neutrophilic asthma phenotype [28]. A plasma protein signature of *α*_2_-macroglobulin, haptoglobin, and hemopexin was shown to discriminate between asthma and COPD with 84% accuracy [29]. This combination of 3 markers was only slightly better than sputum *β*-defensin-1 alone in the current study at 81%. Follow-up studies are warranted to investigate *β*-defensin-1 for its capability for predicting COPD diagnosis and prognosis and future risk of exacerbations.

The epithelium is likely the major source of *β*-defensin-1 that is secreted in the airways. Until recently, only four *β*-defensins (1, 2, 3, and 4), with their genes clustered on chromosome 8, were known to be expressed by epithelial cells [30]. However, additional *β*-defensins are predicted to be expressed, though their biological functions are not clear [9]. Regulation of the expression of *β*-defensin-1 is usually constitutive; however it can be induced in vitro by stimulation with* LPS* and IFN-*γ* [31], poly I:C [32], bacterial components [33], and TNF-*α* and IL-1*β* [34]. *β*-defensin-2 is induced by bacterial products through activation of toll-like receptors (TLRs) or by proinflammatory cytokines including TNF-*α* or IL-1*β* [10]. The expression of *β*-defensins may therefore produce an environment whereby inflammation is enhanced [10], and there is increased vascular permeability [35]. In this way dysregulation of *β*-defensin-1 in severe asthma and COPD is likely to be more harmful than beneficial.

Although the production of *β*-defensins is crucial to proper immune function, altered expression of these molecules may contribute to disease progression. Exposure of the epithelium to cigarette smoke, important in the pathogenesis of COPD, has been shown to influence the production of *β*-defensins [36]. Our study shows that production of *β*-defensin-1 was decreased upon exposure to CSE in healthy pBECs; however, when COPD pBECs were exposed, *β*-defensin-1 production was maintained. This abnormal response of pBECs from COPD patients may be contributing to the higher level of *β*-defensin-1 seen in the sputum, which may be triggered by exposure to cigarette smoke. Other studies have shown that *β*-defensin-1 gene expression is also decreased after CSE exposure in A549 cells, a lung alveolar epithelial cell line [37]. Conversely, other *β*-defensins are upregulated upon smoke exposure [37, 38]. Further investigation is required to understand the mechanisms causing the abnormal *β*-defensin-1 production in COPD.

There are a number of limitations to this study. There needs to be further investigation regarding the stimuli responsible for the elevated *β*-defensin-1 levels in COPD and the mechanisms involved. Future studies should also investigate the relationship of *β*-defensin-1 and bacterial infection. Given the results of the pBEC culture, we would suspect that the epithelial cells are reprogrammed so that their production of *β*-defensin-1 does not alter as it should when exposed to different stimuli. We also did not investigate the relationship of *β*-defensin-1 levels to genotype in this population. Given the associations with DEFB1 polymorphisms and COPD, this should be further investigated. Future studies will need to confirm the biomarker potential of *β*-defensin-1 in a larger population of subjects with COPD, which will also be necessary for detecting associations with disease severity. Further studies should also investigate the levels of *β*-defensin-1 in those patients with asthma COPD overlap.

This study shows that *β*-defensin-1 protein is increased in the airways in COPD and severe asthma but not associated with inflammatory phenotype. In fact, *β*-defensin-1 expression was a strong biomarker for predicting COPD from both asthma subjects and healthy controls. The level of *β*-defensin-1 was correlated with smoking history and, in vitro, *β*-defensin-1 was decreased upon smoke exposure in epithelial cells from healthy participants but maintained in epithelial cells from participants with COPD, suggesting a differential response or tolerance to smoke exposure. This study has identified dysregulated *β*-defensin-1 production, indicating that this protein may be a therapeutic target for COPD and severe asthma.

## Figures and Tables

**Table 1:** Clinical characteristics and sputum cell counts in patients with inflammatory airway diseases compared to healthy controls.

	Healthy controls	Asthma	COPD	*p* value
*N*	28	94	43	
Age (years), mean (SD)	46 (19)	57 (13)	70 (8)^†‡^	<0.001
Gender, M/F	12/16	38/56	23/20	0.356
Atopy, *n* (%)	13 (46)	62 (66)	23 (53)	0.119
FEV_1_, % predicted, mean (SD)	107 (13)	79 (21)^†^	55 (16)^†‡^	<0.001
FEV_1_/FVC, %, mean (SD)	81 (7)	69 (10)^†^	54 (11)^†‡^	<0.001
BMI, kg/m^2^, mean (SD)	26.1 (4)	30.6 (7)^†^	29.3 (7)	0.008
Exhaled nitric oxide (pbb), median (Q1, Q3)	18.6 (14.6, 22.3)	21.6 (14.8, 21.6)	18.1 (14.5, 23.8)	0.127
Smoking, ex/never	9/19	38/56	33/10^†‡^	<0.001
Pack years, median (Q1, Q3)	17 (3, 45)	7 (3, 20)^§^	32 (20, 54)	<0.001
Inhaled corticosteroid (ICS) use, *n* (%)	NA	77 (82)	36 (84)	0.798
ICS dose (*μ*g daily BDP equivalent) median (Q1, Q3)	NA	1000 (500, 2000)	2000 (1000, 2000)	0.012
Long acting *β*-agonist use, *n* (%)	NA	76 (81)	34 (79)	0.808
Long acting muscarinic receptor antagonist (LAMA) use, *n* (%)	NA	13 (14)	24 (56)	<0.001
Total cell count ×10^6^/mL, median (Q1, Q3)	2.5 (1.3, 4.5)^‡§^	3.4 (2.1, 7.1)	4.7 (2.9, 9.9)	0.007
Viability, median (Q1, Q3)	75.0 (64.3, 88.1)	77.6 (68.1, 90.9)	81.5 (75.8, 93.1)	0.142
Neutrophils, %, median (Q1, Q3)	29.0 (10.1, 52.3)	43.8 (24.8, 63.0)^†^	73.1 (46.0, 85.5)^†‡^	<0.001
Neutrophils ×10^4^/mL, median (Q1, Q3)	55.6 (24.5, 134.4)	128.3 (53.5, 324.0)^†^	339.1 (136.6, 705.9)^†‡^	<0.001
Eosinophils, %, median (Q1, Q3)	0.3 (0.0, 0.5)	1.0 (0.3, 4.3)^†^	0.8 (0.4, 2.3)^†^	0.001
Eosinophils ×10^4^/mL, median (Q1, Q3)	0.4 (0.0, 1.9)	5.6 (0.5, 29.8)^†^	6.3 (1.5, 14.4)^†^	<0.001
Macrophages, %, median (Q1, Q3)	66.1 (42.2, 84.0)	43.3 (24.3, 58.5)^†^	18.9 (9.0, 49.3)^†‡^	<0.001
Macrophages ×10^4^/mL, median (Q1, Q3)	150.1 (79.4, 257.5)	137.9 (80.6, 218.7)	111.8 (54.0, 169.7)	0.187
Lymphocytes, %, median (Q1, Q3)	0.9 (0.3, 2.0)	0.5 (0.3, 1.3)	0.3 (0.0, 0.5)^†‡^	0.002
Lymphocytes ×10^4^/mL, median (Q1, Q3)	1.9 (0.4, 4.7)	1.9 (0.2, 5.0)	0.5 (0.0, 1.7)^†‡^	0.009
Columnar epithelial cells, %, median (Q1, Q3)	1.9 (0.5, 7.5)	1.6 (0.3, 4.0)	1.0 (0.3, 2.0)	0.104
Columnar epithelial cells ×10^4^/mL, median (Q1, Q3)	3.1 (1.5, 11.7)	3.7 (1.6, 10.9)	3.4 (1.3, 10.1)	0.959

^†^ *p* < 0.05 versus healthy, ^‡^ *p* < 0.05 versus asthma, and ^§^ *p* < 0.05 versus COPD.

BMI: body mass index; FEV_1_: forced expiratory volume in 1 second; FVC: forced vital capacity; NA: not applicable.

## Abbreviations

AUC: Area under the curve
CA: Controlled asthma
COPD: Chronic obstructive pulmonary disease
CSE: Cigarette smoke extract
DEFB1: *β*-defensin-1
FEV_1_: Forced expiratory volume in 1 second
FVC: Forced vital capacity
GOLD: Global obstructive lung disease
pBECs: Primary bronchial epithelial cells
ROC: Receiver operating characteristics
SA: Severe asthma
TLR: Toll-like receptor
UA: Uncontrolled asthma.
